# The platypus in Edinburgh: Robert Jameson, Robert Knox and the place of the *Ornithorhynchus* in nature, 1821–24

**DOI:** 10.1080/00033790.2016.1230783

**Published:** 2016-09-27

**Authors:** Bill Jenkins

**Affiliations:** ^a^ Science, Technology and Innovation Studies, University of Edinburgh, Edinburgh, Scotland, UK

## Abstract

The duck-billed platypus, or *Ornithorhynchus*, was the subject of an intense debate among natural historians in the late eighteenth and early nineteenth centuries. Its paradoxical mixture of mammalian, avian and reptilian characteristics made it something of a taxonomic conundrum. In the early 1820s Robert Jameson (1774–1854), the professor of natural history at the University of Edinburgh and the curator of the University's natural history museum, was able to acquire three valuable specimens of this species. He passed one of these on to the anatomist Robert Knox (1791–1862), who dissected the animal and presented his results in a series of papers to the Wernerian Natural History Society, which later published them in its *Memoirs*. This paper takes Jameson's platypus as a case study on how natural history specimens were used to create and contest knowledge of the natural world in the early nineteenth century, at a time when interpretations of the relationships between animal taxa were in a state of flux. It shows how Jameson used his possession of this interesting specimen to provide a valuable opportunity for his protégé Knox while also helping to consolidate his own position as a key figure in early nineteenth-century natural history.

## Contents


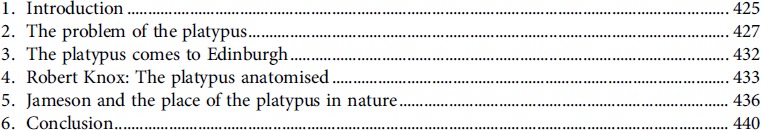


## 1. Introduction

In 1823 Robert Jameson (1774–1854), the University of Edinburgh's professor of natural history, acquired two specimens of the *Ornithorhynchus*, or duck-billed platypus.^1^ 1 In this paper I will refer to the creature as the platypus, as this was the term commonly applied to the animal in the early nineteenth century, as it still is today. These had been sent to Jameson by Sir Thomas Brisbane (1773–1860), Governor of New South Wales from 1821 to 1825, who had himself been a student at the University of Edinburgh in the 1780s. From 1804 until his death in 1854 Jameson was also keeper of the University's natural history museum, usually referred to as ‘Edinburgh College Museum’ or the ‘Royal Museum of the University of Edinburgh’ in this period. Jameson was no stranger to receiving exotic specimens from distant corners of the globe. Nonetheless, he must have been particularly gratified to receive two rare specimens of a species that had been so much talked and written about over previous decades. These were not in fact the first examples of the platypus to arrive at the College Museum in Edinburgh. In 1821 one of the animals was given to the Museum by a donor identified as ‘General Gordon’ in the Museum catalogue.^2^ 2 [William Macgillivray], Catalogue of the Museum of the College of Edinburgh, Natural History Department Registers, Library of the National Museum of Scotland, p. 53. But for a brief note in the catalogue, we know nothing about how this first specimen was received, although it is still to be found in the collections of National Museums Scotland. Since it was first scientifically described by the English zoologist George Shaw (1751–1813) in 1799, this remarkable creature had been the subject of a lively debate among European natural historians. Seeming to combine the characteristics of mammals, reptiles and birds, it was difficult to know where to place it within existing taxonomies. The debate on its place in nature took place at a pivotal time in natural history, when the static, hierarchical taxonomies that had dominated discourse on the natural world in the eighteenth century were being challenged by both the new transcendental approach to comparative anatomy developed in France and Germany and dynamic, evolutionary visions of the history of life on earth, such as those proposed by Jean-Baptiste Lamarck (1744–1829) in France and Erasmus Darwin (1731–1802) in England.

 The debate regarding the place of the platypus in nature in the late eighteenth and early nineteenth centuries has already received significant attention from scholars. Recent studies have taken the form of both general reviews of the controversy and accounts that focus on specific aspects of the anatomy and physiology of the platypus. Ann Moyal has written an excellent book-length general account of the development of scientific discourse on the platypus from its first discovery by Europeans until the present day, and another good general account of the debate is provided by Brian K. Hall.^3^ 3 Ann Moyal, *Platypus: The Extraordinary Story of How a Curious Creature Baffled the World* (Washington, DC, 2001); Brian K. Hall, ‘Thinking of Biology: The paradoxical platypus’, *Bioscience* 49(3) (1999), 211–18. More specialised studies include Peter Hobbins' recent paper, which deals with the role of the venomous spur of the male platypus in the controversy.^4^ 4 Peter Hobbins, ‘A Spur to Atavism: Placing Platypus Poison’, *Journal of the History of Biology* 48 (2015), 499–537. Hobbins includes a very useful account of Robert Knox’s contribution. Another important facet of the debate, in this case the fraught question of whether the platypus laid eggs or gave birth to live young, has been explored in detail by Jacob W. Gruber.^5^ 5 Jacob W. Gruber, ‘Does the platypus lay eggs? The history of an event in science’, *Archives of Natural History* 18(1) (1991), 51–123. Pietro Corsi also makes some perceptive remarks about the important role of Étienne Geoffroy Saint-Hilaire (1772–1844) in the debate. See Pietro Corsi, ‘The revolutions of evolution: Geoffroy and Lamarck, 1825–1840’ (2011), pp. 10–11 http://hsmt.history.ox.ac.uk/staff/documents/Corsi_Lamarckinthe1830s_Oct2011.pdf [accessed 6 May 2014].


My focus in this paper is not on the platypus controversy as a whole, nor upon a particular technical facet of it, but rather upon the arrival of a specimen at a particular site and the activities that followed its arrival. I will put the story of the platypus in Edinburgh in the context of the European-wide debate on its place in the natural order by exploring the views of a variety of leading natural historians. But then I turn to the story of one particular platypus, how it came to be in the hands of Professor Jameson, what its subsequent history was, and what it can tell us about the role of specimens like this in natural historical discourse at that time.

It had always been important for natural historians such as Jameson to build and maintain an extensive network of connections among travellers, collectors and fellow natural historians around the world. The flow of specimens and information they provided was the life blood of natural history. Nicolaas Rupke has shown how Richard Owen (1804–92) created just such a network in his work at the Hunterian Museum in London from 1827 to 1856.^6^ 6 Nicolaas Rupke, *Richard Owen: Biology without Darwin* (Chicago, 2009), pp. 43–52. Owen's own acquisition and dissection of specimens of the platypus in the early 1830s, which he acquired from the collector George Bennett (1804–93), was a significant coup for him. It allowed him to contribute authoritatively to the debate on the place of the animal in the natural order, declaring that the platypus belonged among the mammals, rather than along with the echidna in a class of their own, the Monotremata. Rupke has noted that a ‘major reason for acquiring, displaying, and describing particular specimens was to enhance the ranking of a museum’, and by extension that of its curator.^7^ 7 Rupke (note 6), p. 44. The possession of such specimens also allowed Jameson to pronounce authoritatively to his students and fellow natural historians on one of the most puzzling questions in natural history in the early decades of the nineteenth century, thereby putting him in the company of such exalted figures as Georges Cuvier (1769–1832) and Johann Friedrich Blumenbach (1752–1840), who had also contributed to the debate. Not being a comparative anatomist, he chose not to dissect the creature himself. Instead he passed it on to one of his younger associates who had the necessary technical skills. The professor was the patron of a number of significant younger natural historians in Edinburgh in the 1820s, and it was to one of these, Robert Knox (1791–1862), that he passed one of his precious specimens of the platypus for dissection. Knox subsequently published a number of papers on the beast and its peculiar anatomy in 1824, as well as a further short paper in 1826. In this way Knox too was able to position himself alongside the great figures of comparative anatomy who had already given their opinions on the platypus. As we will see, however, he seems quite deliberately to have done this in a way that did not challenge the authority of the professor. After all, Jameson was also president of Edinburgh's Wernerian Natural History Society, and edited its *Memoirs* as well as the *Edinburgh New Philosophical Journal* (widely known as ‘Jameson’s Journal’). These were the journals in which Knox's papers appeared.

It is well documented that Edinburgh in the 1820s was something of a hotbed of radical ideas about the natural world and humanity's place in it.^8^ 8 See, for example, Adrian Desmond, *The Politics of Evolution: Morphology, Medicine, and Reform in Radical London* (Chicago, IL, 1989) and James A. Secord, ‘Edinburgh Lamarckians: Robert Jameson and Robert E. Grant’, *Journal of the History of Biology* 24 (1991), 1–18. Two of the most radical thinkers in Britain at the time, Robert Edmond Grant (1793–1874) and Robert Knox, both taught in one of the city's extra-mural anatomy schools in this period, where they had every opportunity to promote their unorthodox ideas among Edinburgh's medical students. The city also boasted the Plinian Natural History Society, largely the preserve of students at the Edinburgh medical school, although Grant was also an active member and Knox gave occasional papers to the Society. The Plinian Society provided a forum where ideas such as phrenology, the transmutation of species and Étienne Geoffroy St Hilaire’s new transcendental anatomy were eagerly discussed. Their interest in the theories of Geoffroy was shared not only with Grant and Knox, but also with Robert Jameson, who ‘gave an account of the doctrines of Geoffroy St Hilaire on the analogy between extinct animals and those now living’ to the Wernerian Society on 25 April 1829.^9^ 9 Wernerian Natural History Society, Minutes of the Wernerian Society, 1808–58. 2 vols. University of Edinburgh Library, Dc.2.55, f.297. It is therefore unsurprising that the debate on the taxonomic status of the platypus seems to have aroused lively interest in Edinburgh natural history circles when three specimens of the creature arrived in the city in the early 1820s.

The fate of Jameson's platypus provides a valuable case study on how knowledge of the natural world was created and shared by both the exchange of specimens and by the exchange of knowledge through learned societies and scholarly publications in the early nineteenth century. At a time when understandings of the natural world were shifting and new discourses on the relationships between living things were competing for acceptance, the possession of, and access to, rare and important specimens of this kind provided a significant locus of academic legitimacy and authority.

## 2. The problem of the platypus

In order to understand the problem of the platypus as it appeared to early nineteenth-century natural historians, first we must glance briefly at contemporary mammalian taxonomy, which was very much in a state of flux in the early nineteenth century. There was little general agreement as to exactly how many mammalian orders existed, what these orders were to be called, or how the different species of mammals were to be apportioned among them. I will turn first to the scheme adopted by the great Swedish taxonomist Carl Linnaeus (1707–78), whose model, indicated below, formed the central reference point for all later systems of classification. Although subsequent authorities grouped them differently and moved different types of animals from order to order, they tended to stick to a total of approximately eight orders for the mammals, with the exception of Lamarck.

1. *Primates* (man, apes, monkeys, lemurs and bats)
2. *Bruta* (elephants, manatees, sloths, anteaters and pangolins)
3. *Ferae* (seals, dogs, cats, civets, weasels and their relatives and bears)
4. *Bestiae* (pigs, armadillos, hedgehogs, moles, shrews and opossums)
5. *Glires* (rhinoceros, porcupines, rabbits, beavers, mice and squirrels)
6. *Pecora* (camels, deer, goats, sheep and cows)
7. *Belluae* (horses and the hippopotamus)
8. *Cete* (whales)^10^ 10 Carl Linnaeus, *Systema Naturae per Regna Tria Naturae*, 2nd edn, vol 1 (Stockholm, 1758), pp. 18–19.

The classification schemes adopted respectively by three major authorities in the early decades of the nineteenth century bear witness to the lack of consensus that still reigned at that time. In his *Règne* *Animal* the great French comparative anatomist Georges Cuvier divided the mammals into the following eight orders:

1. *Bimanes* (humans)
2. *Quadrumanes* (apes, monkeys and their relatives)
3. *Carnassiers* (comprising an assortment of mostly carnivorous mammals, from bears and seals to moles and bats, but also including marsupials)
4. *Rongeurs* (rodents and some related forms)
5. *Édentés* (including sloths, armadillos anteaters, pangolins and the *Ornithorhynchus*)
6. *Pachydermes* (elephants, rhinoceros, pigs, horses and their relatives)
7. *Ruminans* (deer, goats, camels and other ruminants)
8. *Cétacés* (whales)^11^ 11 Georges Cuvier, *La Règne Animal, Distribué d’après son Organisation*, vol. 1 (Paris, 1817), pp. 81–287.

To modern eyes many of these divisions may seem quite arbitrary. The ‘Carnassiers’ in particular looks very like a taxonomic rattle bag of different forms with little to connect them in terms of shared morphology.

The German physiologist Johann Friedrich Blumenbach differed from Cuvier in lumping the carnivores, rodents and edentates into one order, the ‘Digitata’, a taxon if anything more disparate than Cuvier's ‘Carnassiers’. As well as putting these three of Cuvier's orders together, he split off the bats and horses and gave each group its own order. He also created an order of web-footed animals, the ‘Plamata’ to include an assortment of amphibious forms.

1. *Bimanus* (humans)
2. *Quadrumana* (apes and monkeys)
3. *Chiroptera* (bats)
4. *Digitata* (animals with separate toes on all four feet, including most of the forms from Cuvier's *Carnassiers*, *Rongeurs* and *Édentés*)
5. *Solidungula* (animals with a single hoof – horses and their relatives)
6. *Bisulca* (corresponding to Cuvier's *Ruminans*)
7. *Multungula* (corresponding to Cuvier's *Pachydermes*, although excluding the horses)
8. *Palmata* (animals with webbed feet, including beavers, seals and the *Ornithorhynchus*)
9. *Cetacea* (whales)^12^ 12 William Lawrence, ‘Introduction’, in J.F. Blumenbach, *A Short System of Comparative Anatomy* (trans. William Lawrence) (London, 1827), pp. xvi–xxii.

Jean-Baptiste Lamarck was best known in this period as Europe's leading invertebrate zoologist, but he did include a table of classification of the mammals in his *Philosophie Zoologique* (1809). His scheme differed radically from those of Cuvier and Blumenbach and contained only four orders (humans are left out of his table and dealt with in a separate section). Lamarck divided the mammals into those with no nails or claws on their feet (the whales), amphibious mammals, mammals with hooves and mammals with claws or nails.

1. *Mammifères exongulés* (whales)
2. *Mammifères amphibies* (seals, dugongs and other aquatic animals)
3. *Mammifères ongulés* (horses, ruminants and pachiderms)
4. *Mammifères onguiculés* (a very large order containing all other mammals, from edentates to apes)^13^ 13 Jean-Baptiste Lamarck, *Zoologie Philosophique*, vol. 1 (Paris, 1809), 343–47. Lamarck did not include the *Ornithorhynchus* among the mammals, but rather regarded them as intermediate forms between birds and mammals.

Although Lamarck's scheme is rather different from those of Cuvier and Blumenbach, he shares with Blumenbach the emphasis he places on the forms of the feet of the different orders of mammals in his scheme of classification. Like Cuvier and Blumenbach he also includes a portmanteau order, the ‘*Mammifères onguiculés*’, which contains an even larger variety of disparate forms than Blumenbach's ‘*Digitata*’. These three schemes of classification give a strong sense of the lack of consensus in the taxonomy of the mammals at this time, with a number of competing and widely divergent models advocated by leading natural historians. As for the platypus, it was not included in Linnaeus' scheme, as he did not know of the animal, Cuvier put it with a selection of other hard-to-place forms in the *Édentés*, Blumenbach found a home for it with the web-footed *Palmata*, while Lamarck excluded it from the mammals altogether.

Given the confusion of different classificatory systems current in this period, it is unsurprising that from its first scientific description in 1799 as enigmatic a creature as the platypus presented natural historians with a puzzle. Although both Cuvier and Blumenbach classified it among the mammals, the animal appeared to combine the characteristics of at least three of the major classes of animals according to the Linnean classification: the mammals, reptiles and birds. Indeed, even before it had been scientifically described, David Collins (1756–1810), Lieutenant-Governor of New South Wales, on seeing a specimen in 1797 noted that it appeared to be ‘an amphibious animal of the mole species’ but that it had ‘instead of the mouth of an animal, the upper and lower mandibles of a duck.’^14^ 14 David Collins, *An Account of the English Colony in New South Wales* (London, 1804), p. 427. George Shaw (1751–1813) of the British Museum, who first described a dried specimen of the platypus and named it *Platypus anatinus* was in no doubt that the animal was a quadruped. Like Collins, however, the presence of a duck-like beak led him to comment ‘Of all the mammalia yet known, it seems the most extraordinary in its conformation; exhibiting the perfect resemblance of the beak of a Duck engrafted on the head of a quadruped.’^15^ 15 George Shaw, ‘The Duck-billed Platypus’, *The Naturalist's Miscellany*, 10 (1799), 228–32 (228). Thomas Berwick (1753–1828) described a second specimen that had been sent to the Literary and Philosophical Society of Newcastle-upon-Tyne by John Hunter, then Governor of New South Wales, in his popular *General History of Quadrupeds* (1800). He declined any attempt to classify the creature, writing that ‘it seems to be an animal *sui generis*; it appears to possess a threefold nature, that of a fish, a bird, and a quadruped, and it is related to nothing that we have hitherto seen’.^16^ 16 Thomas Bewick, *A General History of the Quadrupeds* (Newcastle-upon-Tyne, 1800), p. 521. In 1800 Blumenbach, who had also been able to obtain one of Hunter's specimens from Joseph Banks, named it *Ornithorhynchus paradoxus*. As the generic name *Platypus* had already been given to a genus of beetle, it was Blumenbach's name that stuck, giving it its modern binomial of *Ornithorhynchus anatinus*. Blumenbach himself seems to have been in no doubt that the animal was a member of the Mammalia. Nonetheless, he was aware of the anomalous position of a mammal that seemed to lack the very characteristic which ‘had given rise to the name *mammalia*, by which LINNEUS has distinguished them’.^17^ 17 J.F. Blumenbach, *A Short System of Comparative Anatomy* (trans. William Lawrence) (London, 1807), p. 472. Linnaeus had first introduced the term Mammalia in the tenth edition of his *Systema Naturae* (1758), thereby making the possession of breasts the defining characteristics of this class of animals.^18^ 18 Londa Schiebinger, ‘Why mammals are called mammals: Gender politics in eighteenth-century natural history’, *The American Historical Review*, 98 (1993), 382–411 (382). The apparent absence of them in the female platypus was therefore particularly significant.

The surgeon and anatomist Everard Home (1756–1832) published a description of two specimens of the platypus he had also received from Banks, one male and one female, in the *Philosophical Transactions of the Royal Society* in 1802. Home noted that the excretory and reproductive organs had a common opening, or cloaca, as in birds and reptiles. In his description Home pointed out numerous similarities to the anatomy of birds in the reproductive organs, digestive system and ear of the platypus. He also noted that no evidence of teats could be found in the female. Since the time of Aristotle, giving birth to live young had also been a defining characteristic of mammals. It was therefore a surprise for Home to find that the platypus, in many ways so like a mammal, not only seemed not to produce milk, but had a reproductive system that appeared unsuited to producing live young in the manner of other mammals. From an examination of the female reproductive organs, Home concluded that, like some lizards and dogfish, the platypus was ‘ovi-viviparous’ producing eggs, which then hatched inside the body before birth.^19^ 19 Everard Home, ‘A description of the *Ornithorhynchus paradoxus*’, *Philosophical Transactions of the Royal Society of London*, 92(1) (1802), 67–84 (81–82). In a later paper on the echidna, which he believed to belong to the same genus as the platypus, Home concluded that its unique assemblage of characteristics

… distinguish the Ornithorhynchus, in a very remarkable manner, from all other quadrupeds, giving this new tribe a resemblance in some respects to birds, in others to the Amphibia; so that it may be considered as an intermediate link between the classes Mammalia, Aves, and Amphibia; and, although the great difference that exists between it and the Myrmecophaga [anteaters], the nearest genus we are at present acquainted with, shows that the nicer gradations towards the more perfect quadrupeds are not at present known, the facts which have been stated may induce others to prosecute the inquiry, and render that part of the chain complete.^20^ 20 Everard Home, ‘Description of the anatomy of *Ornithorhynchus hystrix*’, *Philosophical Transactions of the Royal Society of London*, 92 (1802), 348–64 (360–61).

He concluded by suggesting that while the platypus is a quadruped, it cannot be considered a member of the Mammalia.^21^ 21 Home (note 20), 361.


Home did not mean to suggest that the platypus represented an ‘intermediate link’ in a modern, evolutionary sense, but rather that it occupied an intermediate position in the ‘Great Chain of Being’ to which he alludes towards the end of the passage quoted above. It was a commonplace of natural history in the eighteenth century, and into the early part of the nineteenth century, that all living things could be arranged in a continuous chain, with the simplest living monad at the bottom and man at the top. Such an arrangement was felt to manifest the boundless creative power of God. In his famous work on *The Great Chain of Being* (1936) Arthur O. Lovejoy explained the concept of the chain of being in terms of the ‘principle of plenitude’. This encompassed the idea that ‘no genuine potentiality of being can remain unfulfilled, that the extent and abundance of the creation must be as great as the possibility of existence and commensurate with the productive capacity of a “perfect” and inexhaustible Source’.^22^ 22 Arthur O. Lovejoy, *The Great Chain of Being* (Cambridge, MA, 1964), p. 52. For a detailed analysis and critique of Lovejoy's ideas see William F. Bynum: ‘The Great Chain of Being after Forty Years: An Appraisal’, *History of Science*, 13 (1975), 1–28. This concept, combined with the Aristotelian notion that all beings can be arranged in a hierarchy, the ‘principle of gradation’, lead to the conclusion that, according to Lovejoy, all of nature forms a continuous, hierarchical ‘Great Chain of Being’. As the Scottish publisher, natural historian and translator of Buffon, William Smellie (1740–95), put it in his *Philosophy of Natural History*: ‘There is a graduated scale or chain of existence, not a link of which, however seemingly insignificant, could be broken without affecting the whole.’^23^ 23 William Smellie, *The Philosophy of Natural History* (Edinburgh, 1790), p. 520. For Smellie, ‘the gradations from one species to another are so imperceptible that to discover the marks of their discrimination requires the most minute attention.’^24^ 24 Smellie (note 23), p. 55. John Walker (1731–1803), Robert Jameson's predecessor in the chair of natural history at the University of Edinburgh, agreed that ‘[t]here is undoubtedly a continued chain in nature from its lowest subject up to the human species, & which it is to be supposed proceeds from him to his maker, all being linked as in the moral world by the most beautiful & regular gradation’. ^25^ 25 Anon, Notes of John Walker's lectures on natural history (1791), Edinburgh University Library, Dc.10.33, f.37. Living things were locked into an ascending natural order that was complete and unchanging. Given such a scheme, the discovery of intermediate forms in nature was only to be expected. While the platypus might have created a headache for taxonomists, for the more philosophically inclined naturalist it was confirmation of one of the key doctrines of natural theology. As Shaw noted in his *General Zoology* (1800), citing the great French naturalist the Comte de Buffon, with regard to the platypus, ‘whatever was possible for Nature to produce has actually been produced.’^26^ 26 George Shaw, *General Zoology or Systematic Natural History* (London, 1800), p. 232.


There still remained the problem for taxonomists of where exactly to place the platypus in the order of nature. Although Shaw suggested it belonged in the Linnean order Bruta along with the anteater, no clear consensus emerged in the early years of the nineteenth century. In 1803, the French comparative anatomist Étienne Geoffroy Saint-Hilaire, working from the published descriptions, proposed a separate order of mammals, the Monotremata, to accommodate these paradoxical creatures and their relative the echidna.^27^ 27 Étienne Geoffroy Saint-Hilaire, *Catalogue des Mammifères du Museum d’Histoire Naturelle* (Paris, 1803), pp. 222–26. In his description he noted that these animals have a very small caecum, as in birds, and a common cloaca for their excretory and reproductive organs. He also claimed, following the observations of Home, that the females did not produce milk. Geoffroy's colleague at the Paris Museum of Natural History Jean-Baptiste Lamarck (1744–1829) disagreed that the monotremes were mammals. Neither did he think they could be accommodated within the birds or reptiles. Instead he raised the monotremes to the status of a class in their own right. Lamarck interpreted the problematical features of the platypus in terms of his own transformist theory in his *Philosophie Zoologique* (1809).^28^ 28 Jean-Baptiste Lamarck, *Philosophie Zoologique*, 2 vols (Paris, 1809) I, p. 145. We know from a paper he published in 1829 that in 1822 Geoffroy had come round to Lamarck's view that the monotremes merited their own class, stating that ‘it had become necessary to see in them the essence of a new type, to establish for them a *fifth class* among the vertebrate animals’.^29^ 29 ‘qu'il devenait nécessaire de voir en eux l'essence d'un nouveau type, d'établir pour eux une cinquième classe parmi les animaux vertébrés.’ Étienne Geoffroy Saint-Hilaire, ‘Considérations sur des œufs d’Ornithorinque, formant de nouveaux documens pour la question de la classification des Monotrêmes’, *Annales des Sciences Naturelles*, 28 (1829), 157–64 (158). We do not know for certain if news of his change of heart reached Edinburgh before Knox read his papers on the platypus to the Wernerian Natural History in the spring of 1823. If it had, however, as a disciple of Geoffroy who kept in close contact with developments in the French capital, Knox would certainly have been one of the first to know. Geoffroy's colleague and rival at the Museum of Natural History in Paris, the celebrated comparative anatomist Georges Cuvier, preferred to lump the platypus and the echidna in with the armadillos, anteaters and pangolins among the edentates, despite their many unique and bizarre characteristics.^30^ 30 Georges Cuvier, *Le Règne Animal Distribué d’après son Organisation*, 2 vols, (Paris, 1817), II, p. 226. Cuvier kept his mind open with regard to whether these creatures were viviparous or oviparous.

The notable Scottish zoologist and member of the Wernerian Natural History Society John Fleming (1785–1857) gave his opinion of the place of the platypus in nature in the second volume of his *Philosophy of Zoology* (1822). Fleming did not consider that the Monotremata should be placed within the Mammalia, but rather that they constituted a separate taxon of quadruped. However, as he admitted elsewhere in the same book that ‘Species alone, in the divisions of zoology are permanent, all the others being subject to change. The higher divisions are of our own creation, and are altered occasionally, to make way for our increasing knowledge’, too much significance should not be read into the taxonomic position allotted to the Monotremata in Fleming's book. He thought it unwise to give a definitive ruling on whether the animal was viviparous, oviparous or ovi-viviparous. After noting the conflicting opinions of various scholars on the subject he declared that ‘these, however, must be viewed as mere conjectures, scarcely warranted by the appearances of the organs which have been examined.’^31^ 31 John Fleming, *Philosophy of Zoology*, vol. 2 (Edinburgh, 1822), p. 215. He was, however, more certain that the female of the species lacked teats and did not produce milk.

By the early 1820s, it had therefore been established that the platypus represented a curious amalgam of mammalian, avian and reptilian anatomical features. Crucially, however, whether or not the female produced milk or laid eggs had not yet been determined, but was still the subject of lively contention. As a result, there was no consensus as to whether the animals belonged with the mammals or in their own vertebrate class. This was therefore the state of the debate on the place of the platypus in nature as Knox prepared his papers on the dissection of Jameson's specimen for publication in the *Memoirs of the Wernerian Natural History Society*.

## 3. The platypus comes to Edinburgh

While we do not know much about the specimen of the platypus donated by General Gordon in 1821, we know significantly more about the one which arrived in 1823 in the consignment of specimens from Sir Thomas Brisbane. Brisbane took a keen interest in the natural history of the territory he governed and seems to have been in the habit of sending specimens of the local fauna to Jameson.^32^ 32 Henry Lonsdale, *A Sketch of the Life and Writings of Robert Knox the Anatomist* (London, 1870), p. 116. Although Thomas Brisbane had graduated from Edinburgh too early to be a student of Jameson himself, it is likely that the fame of the Museum and its collection would have encouraged him to send specimens back to his *alma mater*. We also know that he had been a non-resident member of the Wernerian Natural History Society since December 1821, and therefore kept in touch with natural history circles in Edinburgh. ^33^ 33 [Robert Jameson], ‘List of Members of the Wernerian Natural History Society of Edinburgh, – continued from Vol. III’, *Memoirs of the Wernerian Natural History Society*, 4 (1823), 586–89 (588). This was also a point of personal contact with Jameson himself, who was the society's founder and perpetual president and edited its *Memoirs*. In an editorial footnote to one of Knox's papers on the anatomy of the platypus in the *Memoirs* Jameson offered the following fulsome tribute to Brisbane as the donor of the specimen:

The specimen of Ornithorhynchus, examined by Dr Knox, was transmitted to the Royal Museum of the University of Edinburgh by His Excellency the Governor of New South Wales, Sir Thomas Brisbane, Baronet. It is highly gratifying to learn, that this distinguished individual is actively employed in forming an extensive collection of the natural productions of the vast country over which he rules; and that the numerous, uncommon, marine animals of the neighbouring coasts and seas, which have so much excited the curiosity of European naturalists, also engage his particular attention, so that, ere long, we trust, many of these will reach our National Museum of Scotland, and thus afford opportunities for interesting investigations and discoveries.^34^ 34 Editor's footnote to Robert Knox, ‘Observations on the organs of digestion and their appendages, and on the organs of respiration and circulation, in the *Ornithorhynchus paradoxus*’, *Memoirs of the Wernerian Society*, 5(1) (1824), 144–50 (144).

Jameson seems to have had no difficulty in soliciting specimens from both former students and fellow natural historians. Indeed, it appears he was so inundated with items for the Museum that the space it had available for their display and storage had become quite inadequate by the mid-1820s. Jameson told the Scottish Universities Commission of 1826 that ‘a considerable proportion of the recent additions to the Museum have been the contributions of former students’.^35^ 35 Scottish Universities Commission (1826), *Evidence, Oral and Documentary, Taken and Received by the Commissioners Appointed by His Majesty George IV., July 23d, 1826; and Re-appointed by His Majesty William IV., October 12th, 1830; for Visiting the Universities of Scotland. Volume 1. University of Edinburgh* (London, 1837), p. 142.


Thanks to the meticulous record keeping of Jameson's assistant, William MacGillivray (1796–1852), we know that Brisbane's two platypus specimens arrived on the evening of Saturday, 29 March 1823, along with an assortment of other material.^36^ 36 William MacGillivray, Weekly Report Book of the Museum of the University of Edinburgh, vol. 1, from 18th March, 1822 to 19th July 1823, Natural History Department Registers, Library of the National Museum of Scotland, 185–86. MacGillivray remarked that the specimens of the platypus were ‘in bad condition, the rostrum half dissolved, and the pile loose, which is always the case when quadrupeds are sent in spirits.’^37^ 37 MacGillivray (note 36), 156. As well as the keg containing the platypuses, Brisbane had sent the skull of a seal, a piece of gypsum from Macquarie Island, a ‘small box containing pebbles and sand’, an elephant tusk, the head of a Maori, and skins of ‘the native bear of N.S. Wales’, ‘the Flying Squirrel’ and a seal. The fame of Edinburgh's natural history museum was such that new specimens to add to the collection regularly arrived addressed to Jameson. The boxes and kegs from Brisbane were the fourteenth of 37 consignments of specimens to arrive at the Museum that year, many of them consisting of multiple items. According to the catalogue of the Museum, which survives from 1820 onwards, it received consignments of new objects at the rate of approximately one a week in the 1820s. Given Jameson’s policy of never refusing objects offered to him, because refusing them ‘might deter others from sending what was valuable’, it is not surprising that finding space for all these specimens in the Museum rapidly became a problem.^38^ 38 Scottish Universities Commission (1826), *Evidence, Oral and Documentary, Taken and Received by the Commissioners Appointed by His Majesty George IV., July 23d, 1826; and Re-appointed by His Majesty William IV., October 12th, 1830; for Visiting the Universities of Scotland. Volume 1. University of Edinburgh* (London, 1837), p. 142.


Although Jameson clearly actively encouraged students, present and former, and other contacts around the globe to send him objects, there is little evidence that he actively solicited particular specimens. Jameson was not, therefore, assembling his collection ‘as a self-aware process of creating a set of objects conceived to be meaningful as a group’, as Sharon Macdonald has suggested is generally a defining feature of museum collections.^39^ 39 Sharon Macdonald, ‘Collecting practices’, in *A Companion to Museum Studies*, ed. by Sharon Macdonald, (London, 2006), p. 82. Although once in the Museum the specimens seem to have been arranged and displayed in a manner consonant with contemporary ideas of the order of the natural world, the actual acquisition of objects depended largely on the vagaries of the donors and did not represent a consistent collecting programme. Even the consignment of objects of which the platypus formed a part included an odd assortment of mineralogical specimens and a human head as well the bones and skins of other animals.

## 4. Robert Knox: The platypus anatomised

Only one of the specimens of the platypus sent by Brisbane joined the collection in the Museum immediately. The other was given for dissection to Robert Knox, who at this time taught anatomy at the extra-mural anatomy school run by John Barclay (1758–1826) in Surgeon's Square, near the University. At this period Knox seems to have been a close associate of Jameson. He has been seen by some scholars as an expounder of fiercely radical views on the natural world at this time, although there has been little consensus on what exactly his views were. Adrian Desmond has painted a picture of Knox as an anti-progressive transformist, while Philip F. Rehbock considered on the contrary that in his writings on the nature of genera and species of animals he ‘was not thinking of an evolutionary process’.^40^ 40 Desmond, (note 8), 50. Evelleen Richards, on the other hand, has asserted ‘[t]hat Knox held to a theory of organic descent is beyond question’.^41^ 41 Evelleen Richards, ‘The "Moral Anatomy" of Robert Knox: The Interplay between Biological and Social Thought in Victorian Scientific Naturalism’, *Journal of the History of Biology*, 22 (1989), 373–436 (399). Richards' opinion certainly seems in accordance with some of the pronouncements on the transmutation of species that are scattered throughout Knox's later work; for example, in a book published in 1855 he boldly stated that ‘I believe all animals to be descended from primitive forms of life’.^42^ 42 Robert Knox, ‘Enquiries into the philosophy of zoology. Part 1. – On the dentition of Salmonidae’, *The Zoologists: A Popular Miscellany of Natural History*, 13 (1855), 4789–90. However, these transformist assertions all come from the 1840s and 50s, decades after 1823. In fact we have no evidence of any explicit reference to transformism from Knox in the 1820s. In particular, he seems to have been rather circumspect in the views he was prepared to express in print, where there is little trace of the fiery radical depicted by some scholars.

According to Henry Lonsdale, Knox's biographer and former student, his ‘studies at the Museum of Natural History made him known to Professor Jameson, who was glad to receive the aid of a promising naturalist for his *Quarterly Philosophical Journal* [sic]’.^43^ 43 Lonsdale (note 32), p. 36. Lonsdale incorrectly remembered the title of Jameson's journal in this quotation; it was in fact entitled the *Edinburgh Philosophical Journal* rather than the *Quarterly Philosophical Journal*. Jameson had invited Knox to join the Wernerian Society in early 1821, not long after the latter's return from the Cape, where he had served as a military surgeon. Knox read his first paper, on a ‘Caffre albino’, to the Society on 21 March of that year.^44^ 44 [Robert Jameson], ‘Proceedings of the Wernerian Natural History Society’, *Edinburgh Philosophical Journal*, 6 (1821–22), 172–73 (172). Although he left to study in Paris soon after, only returning in December 1822, on his return he became very much a central figure in Edinburgh natural history circles and a regular contributor to the Wernerian Society and the journals edited by Jameson. While Jameson was essentially a mineralogist, Knox was a skilled and experienced comparative anatomist who had studied his craft at the famous Museum of Natural History in Paris, where he was deeply influenced by the work of the great comparative anatomists Étienne Geoffroy Saint-Hilaire, Georges Cuvier and Henri Marie Ducrotay de Blainville.^45^ 45 A.W. Bates, *The Anatomy of Robert Knox: Murder, Mad Science and Medical Regulation in Nineteenth-Century Edinburgh* (Brighton, 2010), pp. 44–47. It was therefore very convenient for Jameson to be able to call on the services of Knox to dissect the valuable specimen that had just arrived from New South Wales. Jameson regularly passed interesting specimens on to Knox to dissect at around this time, the latter having reason to gratefully acknowledge his benefactor in print for this on at least five occasions between 1824 and 1826.^46^ 46 See Knox, ‘An account of the *Foramen centrale* of the retina generally called the *Foramen of Soemmering*, as seen in the eyes of certain reptiles’, *Memoirs of the Wernerian Society*, 5(1) (1824), 1–7; Knox, ‘Inquiry into the origin and characteristic differences of the native races inhabiting the extra-tropical part of southern Africa’, *Memoirs of the Wernerian Society*, 5(1) (1824), 206–19; Robert Knox, ‘On the Wombat of Flinders’, *Edinburgh New Philosophical Journal*, 1 (1826), 104–12; Knox, ‘Observations on the duck-billed animal of New South Wales, the *Ornithorynchus paradoxus* of naturalists. Memoir I. On the organs of sense, and on the anatomy of the poison gland and spur’, *Memoirs of the Wernerian Society*, 5(1) (1824), 26–41; and Robert Knox, ‘Notice respecting the presence of a rudimentary spur in the female echidna of New Holland’, *Edinburgh New Philosophical Journal*, 1 (1826), 130–32. As MacGillivray commented in the Museum's Weekly Report Book on receiving the specimens, preservation in spirits had resulted in the specimens arriving in a poor state. Nevertheless, it seems to have preserved the animal well enough for Knox to attempt a comprehensive dissection, although he reported that in particular the eyes and kidneys had suffered from their long journey and the manner of preservation. He described how the latter ‘had suffered so much by long maceration in spirits, that they were reduced to a pulpy, homogenous mass, which gave way under the slightest pressure. Nothing could be made of their internal structure.’^47^ 47 Robert Knox, ‘Memoir III. On the kidneys, urinary bladder, and organs of generation, in the male of the *Ornithorhynchus paradoxus*’, *Memoirs of the Wernerian Society*, 5(1) (1824), 151– 60 (151).


Before presenting his findings to the more august audience of the Wernerian Natural History Society, Knox first gave a series of papers on 20 April 1823 to the Plinian Natural History Society, which had been founded earlier that year by and for students of Robert Jameson's natural history class. Unfortunately we know nothing of the content of these papers or whether they were identical to the memoirs subsequently given to the Wernerian Society.^48^ 48 Plinian Natural History Society, Abstract of the proceedings of the Plinian Society from its first meeting Jan 14, 1823, to July 25, 1826, Session II. 1823–4, Centre for Research Collections, Edinburgh University Library, SD 6944/1, p.13 Knox then gave a series of four memoirs on the platypus in meetings of the latter Society in April and May 1823. Three of these, covering most of the anatomy of the creature, were read to the Society on 26 April, while the final one, on the anatomy of the animal's poison-gland and spur and sense organs, was read on 17 May. Knox presented his findings to the Society with the help of a series of anatomical illustrations, reproduced in the published versions of the memoirs. He also presented the creature's skeleton for the consideration of the Society's members. Knox's papers on the platypus were then published in the *Memoirs of the Wernerian Society* the following year, complete with a fulsome tribute to Jameson, ‘the distinguished naturalist who intrusted me with the dissection, and who, now for a long time, with unexampled liberality, has forwarded, to the utmost of his power, my researches in comparative anatomy.’^49^ 49 Robert Knox (note 47), 158 The first of these papers, entitled ‘Observations on the Anatomy of the Duckbilled Animal of New South Wales, the Ornithorhynchus paradoxus of Naturalists: Memoir I. On the organs of sense, and on the anatomy of the poison-gland and spur’, was in fact the last to be read to the Society. Knox noted in the introduction that it was Jameson ‘who did me the honour to entrust the dissection to me’, but that the completeness of his dissection was limited by the need to ‘respect the skeleton, which is intended for the Museum’.^50^ 50 Robert Knox, ‘Observations on the anatomy of the duckbilled animal of New South Wales, the *Ornithorhynchus paradoxus* of naturalists: Memoir I. On the organs of sense, and on the anatomy of the poison-gland and spur’, *Memoirs of the Wernerian Society*, 5(1) (1824), 26–41 (27).


Knox observed that, while a significant number of specimens of the platypus had been dissected before in France and Germany as well as by British anatomists, ‘the descriptions of the most celebrated anatomists were completely at variance with each other, and with those of naturalists, relative to the anatomy of some very important organs’. He therefore hoped that his work would help to resolve some of these contradictions.^51^ 51 Knox (note 50), 27 Hobbins comments that Knox ‘belittled his predecessors’ dissections' in his memoirs, perhaps with the aim of emphasising the greater rigour of his own work.^52^ 52 Hobbins (note 4), 509. Although Knox considered that Cuvier was more correct to place the monotremes with edentates than Blumenbach, who placed them with the beaver, seal and manatee, he was aware that the female seemed not to produce milk and that therefore some naturalists, such as Geoffroy, had questioned whether they should be included in the mammals at all. Citing Home’s paper in the *Philosophical Transactions of the Royal Society*, he confidently stated that the animal was oviparous.^53^ 53 Although, as we have seen above, Home actually believed the Ornithorhynchus to be ovoviviparous. He then proceeded to give a detailed account of his dissection of the sense organs and the remarkable venomous spur of the male animal, illustrated with a figure showing its anatomical structure. The second, third and fourth memoirs dealt respectively with the organs of digestion, respiration and circulation, urinary and sexual organs, and the skeleton, musculature and nervous system. Having promised to return the skeleton of the creature to Jameson for the Museum, Knox was unable to examine organs, such as the brain, which would have required damaging bones to reach.

Knox thought the similarity between the sexual and excretory organs and those of birds had been exaggerated by earlier investigators, particularly in France; indeed he claims that they are closer to the arrangement found in the beaver than to that of a bird.^54^ 54 Knox (note 47), 153. He did, however, acknowledge that its skeleton exhibited some decidedly reptilian characteristics, confirming the paradoxical nature of the beast.^55^ 55 Robert Knox, ‘Memoir IV. On the osseous, muscular and nervous systems of the *Ornithorhynchus paradoxus*’, *Memoirs of the Wernerian Society*, 5(1) (1824), 161–74 (161). Elsewhere Knox also admitted that in some details of its skeletal structure the animal presented ‘a compound of the three classes Mammalia, Aves and Reptilia.’^56^ 56 Knox (note 55), 165. In summary, Knox, while acknowledging its reptile affinities, downplayed the bird-like attributes of the platypus noted by other anatomists, observing that ‘the analogy supposed to exist between the platypus and Birds is reduced to the resemblance of the ossicula of the ear, and to the female organs of generation, which I have not as yet had an opportunity of examining.’^57^ 57 Knox (note 55), 172. Although he acknowledged that some authorities considered the platypus to belong in a distinct fifth class of vertebrates, Knox stopped short of endorsing this view himself in his published memoirs on its anatomy. His reticence on the subject might seem at odds with the boldly speculative writings of his later years. However, at this juncture in his career it might have served him better to avoid controversy and maintain a more measured, descriptive approach to the strange anatomy of the platypus, at least in his published work. The measured, deferential tone of his memoirs, especially with reference to Jameson, might well reflect this deliberately cautious approach. As we will see below, Jameson did pronounce definitely on the affinities of the platypus to his students, therefore it is plausible to suggest that Knox refrained from doing so out of respect for his benefactor. The dissection completed and written up, true to his word, Knox returned the skeleton to the Museum, where MacGillivray dutifully noted that the ‘skeleton of the platypus dissected by Dr. Knox has been deposited in the British Gallery.’^58^ 58 William MacGillivray, Weekly Report Book of the Museum of the University of Edinburgh, Vol. 1, from 18th March, 1822 to 19th July 1823, Natural History Department Registers, Library of the National Museum of Scotland, 228. Knox's involvement with the platypus did not in fact end here. In 1826 he published a description of the rudimentary spur of a female specimen of a platypus provided by Robert Jameson in the *Edinburgh New Philosophical Journal*, a periodical edited by Jameson. See Robert Knox, ‘Notice respecting the presence of a rudimentary spur in the female echidna of New Holland’, *Edinburgh New Philosophical Journal*, 1 (1826), 130–32.


## 5. Jameson and the place of the platypus in nature

**Figure 1.**
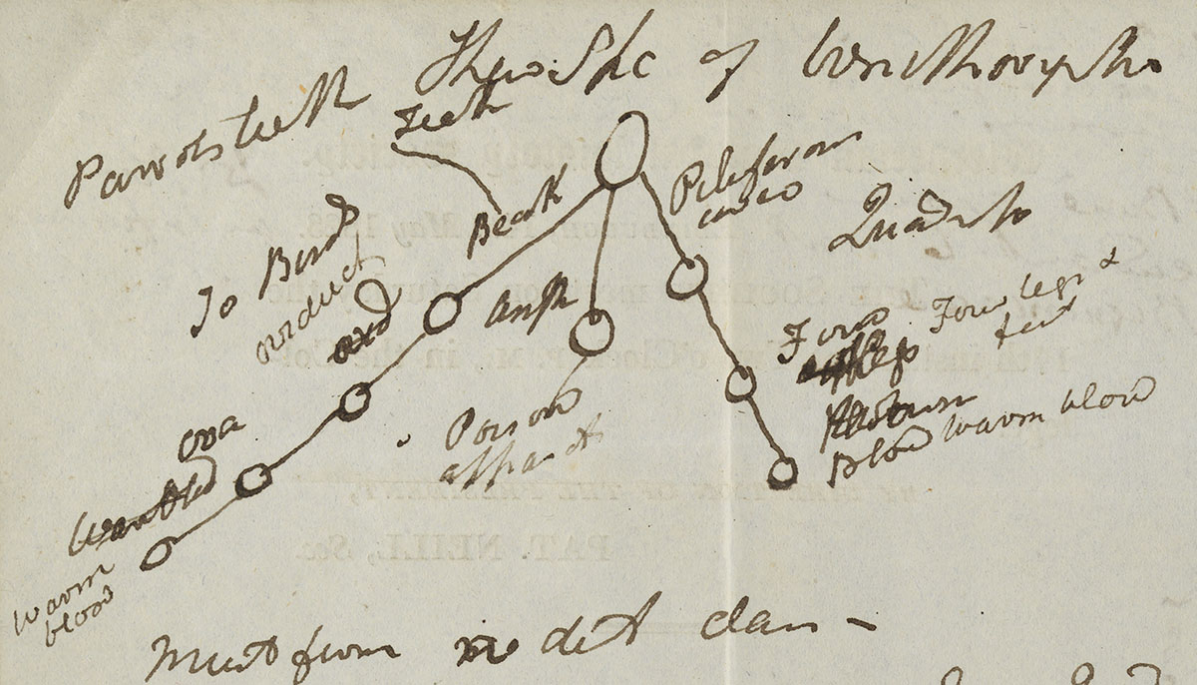
Diagram showing the relationship of the monotremes to other vertebrate taxa, drawn by Robert Jameson on the reverse of the programme for a meeting of the Wernerian Natural History Society dated 17 May 1823 (Source Jameson (note 62), Edinburgh University Library CC-BY).

Even before receiving the specimens from Brisbane, Jameson seems to have followed Geoffroy and Lamarck in placing the monotremes in their own order of vertebrates, falling between reptiles (classed under Amphibia by many natural historians in Jameson’s day), birds and mammals, but distinct from any of them. In his lectures in 1822, Jameson told his students that they are ‘a link between the Mammalia, Amphibia & Aves’ and ‘may form a Class’.^59^ 59 Robert Jameson, zoology lectures, 1822 (notes taken by George Gordon), Elgin Museum, L1987.5.3 (28/5), f.48 recto. To say that a species constituted a link between different taxonomic groups in the 1820s could be interpreted in at least three different ways. Firstly, it could suggest that it formed a hitherto missing link in the chain of being. This would then be a late manifestation of an essentially eighteenth-century vision of the natural world. Secondly, it could refer to the relationships between living things implied by the new transcendental comparative anatomy of Geoffroy and his disciples, which assumed that all animals, or at least all vertebrates, were variations on a single, archetypal design. It would therefore be expected that intermediate forms would exist uniting the different groups of animals, as they were all variations of the same fundamental body plan. Richard Owen, who championed a specifically English, Deistic form of transcendental anatomy in the 1830s and 40s, considered that the creative power of the deity would have realised every potential variation of the common vertebrate plan, and worried that the ‘conceivable modifications of the vertebrate archetype are very far from being exhausted by any of the forms that now inhabit the earth, or that are known to have existed here at any period.’^60^ 60 Richard Owen, *On the Nature of Limbs* (London, 1849), p. 83. He resolved this apparent problem by proposing that the other possible variations on the common theme might exist on other planets. To those who understood the natural world in this way, apparently intermediate forms such as the platypus were more than welcome as proof that all possible variations of the archetype had indeed been brought into existence. Thirdly, the intermediate position of the platypus between mammals, birds and reptiles could be taken as evidence of an evolutionary relationship between these major groups of land vertebrates. This transformist reading is in fact quite compatible with that based on transcendental anatomy, and Geoffroy himself certainly came to combine the two from the early 1820s onwards, regarding the archetype not just as an ideal form but as an ancestral animal that had actually existed as a living creature. This is the interpretation that would have been obvious to transformist thinkers such as Lamarck and Geoffrey, at least after the latter's conversion to transformism.^61^ 61 Geoffroy's first openly transformist work was his ‘Recherches sur l’organisation des gavials ; Sur les affinités naturelles desquelles résulte la nécessité d’une autre distribution générique, *Gavialis*, *Teleosaurus* et *Steneosaurus* ; et sur cette question, si les Gavials (*Gavialis*), aujourd’hui répandus dans les parties orientales de l’Asie, descendent, par voie non interrompue de génération, des Gavials antidiluviens, soit des Gavials fossiles, dits Crocodiles de Caen (*Teleosaurus*), soit des Gavials fossiles du Havre et de Honfleur (*Stenosaurus*)’, *Memoires du Muséum d’Histoire Naturelle*, 12 (1825), 97–155. In which way is Jameson's terse statement on the relationship between the platypus and the major groups of land vertebrates then to be interpreted? A diagram scribbled by Jameson on the back of the agenda for a meeting of the Wernerian Society held on 17 May 1823 may shed some further light on how he envisaged this relationship (see Figure 1).

Jameson's diagram shows a single unlabelled circle, from which emerge three lines diverging downwards, each with a series of smaller circles strung out along it like beads on a string. The line on the left is labelled ‘To birds’, with some of the characteristics that birds share with the platypus written beside each of the smaller circles, including ‘beak’, eggs (‘ova’), a characteristic form of ‘oviduct’ and ‘warm blood’. The line going to the right is labelled ‘Quadrup[eds]’ and has the characteristics that mammals share with the platypus written beside each of the three smaller circles: fur (‘piliferous cover’), ‘four legs & feet’ and ‘warm blood’. The shortest, middle line, only has a single circle at its end and is labelled ‘Amph[ibians]’, the latter including creatures that would now be classified as reptiles. Beneath it is written ‘poison apparat[us]’, referring the venom glands of some reptiles, which Jameson seems to have identified with the venomous spur of the male platypus. Beneath the diagram is written:

Must form dif. class.

1. There is resemblance to Birds, Quad & Amphibia.

2 But it cannot be arranged with any of these & must form a member of a new class.^62^ 62 Robert Jameson, handwritten notes on the back of a Wernerian Historical Society meeting agenda, Centre for Research Collections, Edinburgh University Library, gen. 129.

Jameson seems here once again to have been following the opinion of Lamarck and Geoffroy that the platypus represented a separate class, with clear affinities with birds, mammals and reptiles, but belonging to none of these. But would Jameson have agreed with Lamarck and Geoffroy that these shared characteristics represented evolutionary relationships between these different vertebrate classes? To modern eyes, this diagram would seem transparently to represent an evolutionary tree of life of a kind that is now very familiar. As it has been suggested elsewhere, notably by James Secord, that Jameson was at least sympathetic to transformist interpretations of the history of life, if not actually a fully-fledged transformist himself, an evolutionary interpretation should not be dismissed out of hand.^63^ 63 Secord (note 13), 1–18. See also Bill Jenkins, ‘Neptunism and transformism: Robert Jameson and other evolutionary theorists in early nineteenth-century Scotland', *Journal of the History of Biology* 49 (2016), 527–57. It seems more than likely that Jameson jotted down his diagram while listening to Knox give his paper before the Wernerian Society, or shortly afterwards, so it is probable that it reflects the opinions of Knox as well as those of Jameson.

**Figure 2.**
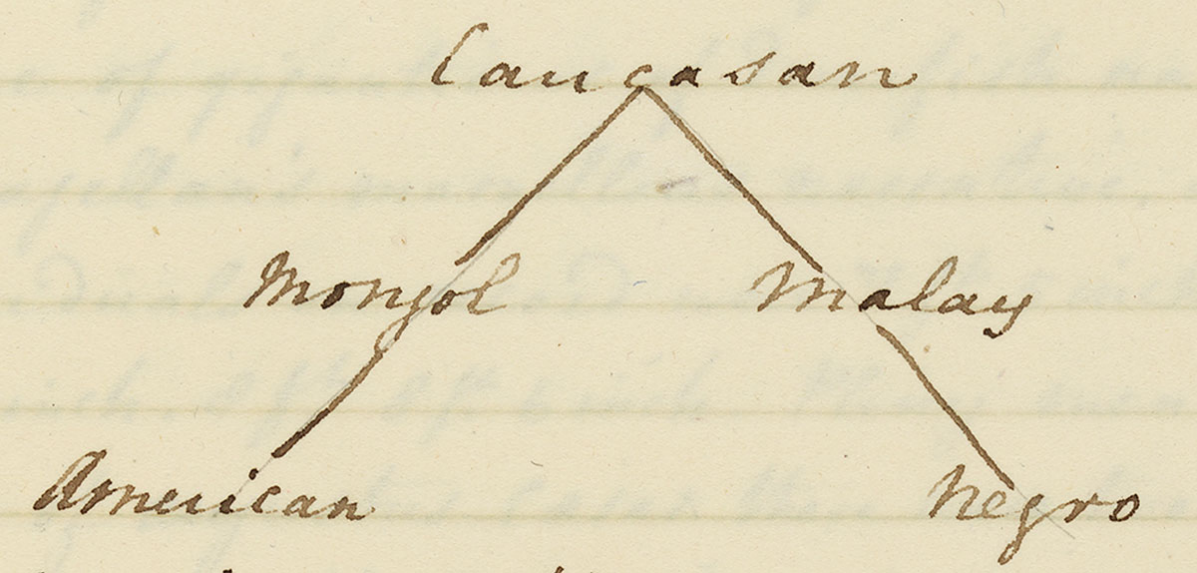
Diagram showing the relationships between the races of man, from an undated set of student notes taken in one of Robert Jameson’s natural history lectures by an anonymous student (Source Jameson (note 64), Edinburgh University Library CC-BY).

An evolutionary interpretation might seem at first glance to be confirmed by a very similar diagram to be found in the notes taken by a student in one of Jameson's natural history lectures. The subject under discussion here was the races of man, and the student had copied down a diagram representing the relationship between the races (see Figure 2). The student has written above the diagram that ‘It is supposed that the Caucasian is the original stock whence all the varieties have sprung.’^64^ 64 Robert Jameson, zoology lectures (notes taken by an anonymous student), vol. 2 (n.d.), Centre for Research Collection, Edinburgh University Library Dc.2.34, f.237. At the head of the family tree is the Caucasian race, with two branches of the human family descending from it, one leading to the American race by way of the Mongol race, and the other leading to the Negro race via the Malay race. This is essentially the genealogy of the races proposed by Blumenbach, and there is no doubt that this diagram is intended to be a genealogical tree, although it is important to note that the process illustrated in the diagram of the relationship between the races is one of degeneration, not of evolutionary progress. This might seem to suggest that Jameson's very similar diagram of the relationships between the major groups of land vertebrates and the platypus could also be read as representing genealogical relationships.

**Figure 3.**
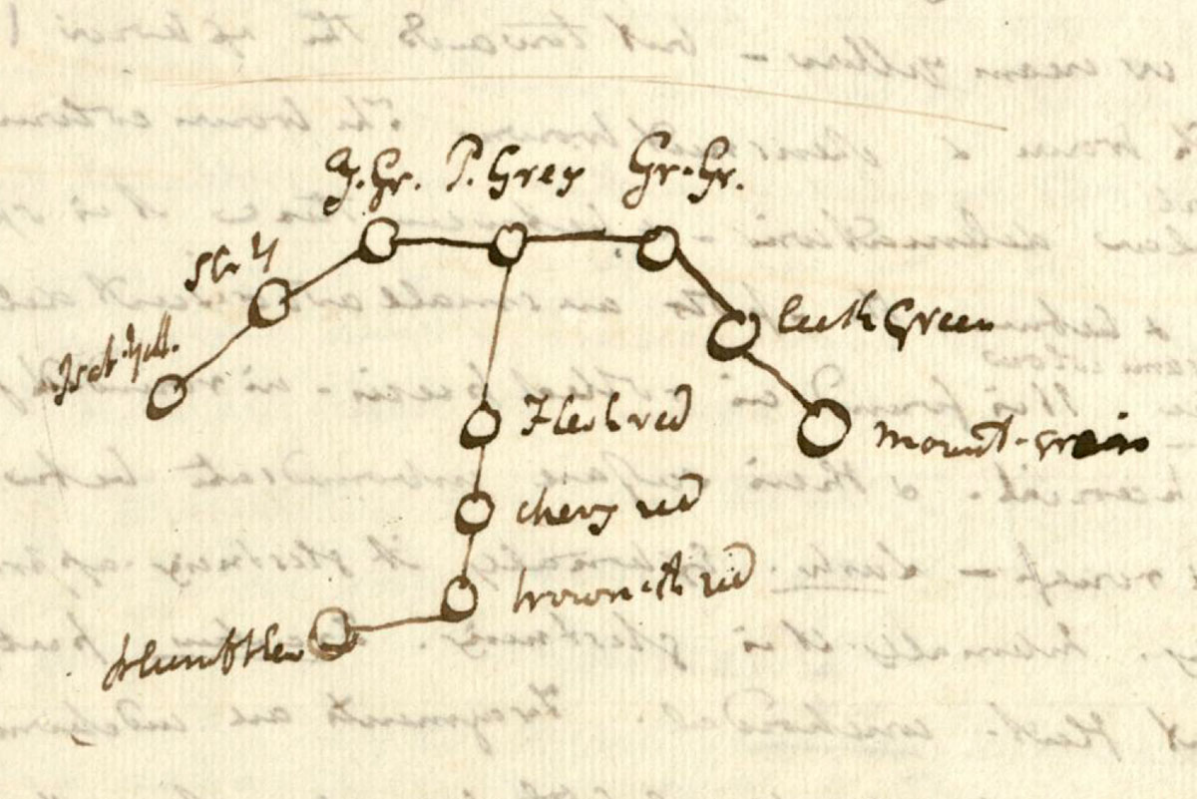
Diagram of the different colours of the semi-precious stone jasper by Robert Jameson, from an undated mineralogical manuscript (Source Jameson (note 66), Edinburgh University Library CC-BY).

Tree diagrams, however need not always suggest an evolutionary relationship; resolutely non-transformist thinkers since at least the mid-seventeenth century had speculated that the relationships between living things could best be described not as a chain but a branching tree, without any implication of a genealogical connection between taxa. Peter Simon Pallas (1741–1811), for example, proposed just such a model in his *Elenchus Zoophytorum* (1766).^65^ 65 Mark A. Ragan, ‘Trees and networks before and after Darwin’, *Biology Direct*, 4(43) (2009). There also exists another superficially very similar diagram by Jameson, which clearly does not represent any kind of genealogical relationship (see Figure 3). If we look at this diagram, which comes from a manuscript found among Jameson's papers, we can see an example of the same style of diagram being used by Jameson for a very different purpose.^66^ 66 Robert Jameson, untitled mineralogy manuscript (n.d.), Centre for Research Collections, Edinburgh University Library, gen. 122, f.8 verso. This diagram shows the different colours found in the semi-precious stone jasper. The labels are abbreviated and not always easy to read, but we can see from them and the accompanying text that the central blob represents ‘pearl grey’, while, for example, the middle branch represents a continuum passing from there to ‘flesh red’, to ‘cherry red’, then to ‘brownish red’, and finally to ‘plum blew [blue]’. This diagram clearly represents a classification of the different colours of jasper, and there is no suggestion that these relationships are to be read as genealogical, either in the diagram itself or in the accompanying text.

I would argue that in reality Jameson's platypus diagram represents neither a genealogical relationship, such as the one between the races of man, nor a simple taxonomy of the type represented by the tree diagram of the different colours of jasper. Instead I would suggest that it simply provides a graphic representation of the distribution of characteristics shared by the platypus with three of the major classes of vertebrates. Each circle on the line represents one essential characteristic shared with one of the three classes; four with the birds, one with the amphibians and three with the quadrupeds. It therefore illustrates the contention that Jameson shared with Lamarck and Geoffroy that the platypus does not belong to any single one of these vertebrate classes, sharing as it does characteristics with all of them, but must be placed in its own class. It is clear that Jameson habitually used this form of tree diagram to indicate a variety of types of relationships between entities in the natural world. Given that the same type of branching diagram could be used for such different purposes, their interpretation becomes decidedly uncertain. However, it seems apparent in this case that the diagram was simply an aid for Jameson in conceptualising the place of the platypus in nature, as indicated by the characteristics it shared with the major vertebrate classes. It is, however, significant that Jameson chose to indicate the place of the platypus in nature through a diagram showing the distribution of characteristics across a branching network, rather than the tabular representation familiar from most natural history treatises of the period. Instead of showing it occupying a place in a hierarchy, it represents the platypus as a node in a network of characteristics found among different taxa of vertebrate animals. This is a long way from the linear vision of the natural world as a continuous chain passing from higher to lower forms that was evident in the lectures of Jameson's predecessor Walker. How far Jameson had moved away from this model is illustrated in a set of student notes from one of his lectures taken in 1816/17. Here Jameson remarks that some scholars

have maintained that there is in the universe a complete gradation in the scale of beings, that all substances are bound together by certain links and that there is a regular chain of succession in the order of existences from man even to inert stone. But all this opposition is founded on premises unwarrantable and theories perfectly illusory. There is in truth no such regular gradation; and there are wanting many links to connect the whole. It is obvious to all, that if once the chain be broken, this amusing system is overthrown.^67^ 67 Robert Jameson, zoology lectures (notes taken by an anonymous student), 2 vols (1816/17), Centre for Research Collection, Edinburgh University Library Dc.10.32, f.1.

The diagram cannot provide evidence to indicate Jameson's opinions regarding the transformist speculations that Secord and Desmond have demonstrated were current in Edinburgh in the 1820s. However, the references to the position of the platypus in a distinct fifth class of vertebrates here and in his lectures does clearly put him in the same camp as Lamarck and Geoffroy as regards the relationship to the established vertebrate classes. The platypus and its relative the echidna were at once distinct from and intermediate between the existing classes of terrestrial vertebrates, occupying a position in the order of nature mapped out by a unique constellation of vertebrate characteristics.

## 6. Conclusion

We have seen how Jameson sided with the French transformist thinkers Lamarck and Geoffroy in the debate regarding the place of the platypus in nature that was taking place in the early 1820s, preferring to place it alongside the echidna in a class of their own rather than seeing them as representing an aberrant order of the class Mammalia. However, there is no evidence here that he shared their vision of a relationship of common descent between the vertebrate classes. In any case, the evidence suggests that he was firmly of the opinion that the platypus was not a member of any of the established vertebrate orders, but represented at the same time both an intermediate form and a distinct order. Its unique combination of reptilian, avian and mammalian characteristics mapped out a unique place for the monotremes in the natural order. In this he had left the Great Chain of Being far behind, replacing it with a far more flexible model of relationships between taxa.

Whatever conclusions Jameson drew on the taxonomic status of the platypus, the acquisition of three specimens of the platypus in the early 1820s could only have enhanced the reputation of the collection, and thus his own. As well as providing another star exhibit for the Museum, having three specimens of the animal to hand meant he could spare one to pass on to Knox for dissection. The account of this dissection would then be read before the Wernerian Society, which was largely Jameson's creation, and published in its *Memoirs*, with fittingly effusive tributes not only from Jameson to Brisbane, but also from Knox to Jameson. The Society itself, and hence its president, could also only gain in prestige from participation in the high profile and decades-long debate on the true nature and affinities of the platypus. Once Knox had finished his dissection, Jameson would have the skeleton back to add a further important specimen to the Museum's extensive collection.

Knox also clearly stood to benefit from being the recipient of Jameson's patronage. He gained the fame of being one of the relatively few comparative anatomists to have conducted a dissection of the animal. As a teacher in John Barclay's extra-mural anatomy school, Knox relied on the fees of paying students. To be entrusted with the dissection of a platypus was an obvious tribute to his standing as a skilled comparative anatomist. It reflected not only his highly developed technical skills in the art of dissection, but his wide knowledge of the rival doctrines of comparative anatomy in what was at the time a dynamic and exciting field of study, prone to rapid development and fierce antagonism between the champions of rival theoretical approaches.^68^ 68 A famous example of such a controversy was the acrimonious debate that broke out in 1830 between Georges Cuvier and Geoffroy Saint-Hilaire, both of whom had previously commented publicly on the place of the platypus in nature. For a detailed account of this fascinating episode, see Toby A. Appel, *The Cuvier-Geoffroy Debate: French Biology in the Decades before Darwin* (New York, 1987). His anatomy class was already the largest of any of the extra-mural schools in Edinburgh, and the added publicity that his dissection of the platypus brought him could certainly do him no harm. Through the papers he presented to the Plinian Society and the Wernerian Society he enlisted both students and fellow naturalists as ‘virtual witnesses’ to his virtuoso dissection.^69^ 69 For a classic account of the idea of ‘virtual witnessing’, see Steven Shapin and Simon Schaffer, *Leviathan and the Air Pump: Hobbes, Boyle, and the Experimental Life* (Princeton, NJ, 1985), pp. 60–65. Knox seems to have been relatively circumspect with regard to the possible status of the monotremes as a fifth vertebrate class, at least in his published work. We know that Jameson told his natural history class that the platypus belonged to a new class of animals, sharing features with mammals, birds and reptiles, but we do not know if Knox shared his opinion. While he acknowledged that some authorities considered that they represented a new class of vertebrates, he did not venture his own opinion as to the correct classification of the creature when he published the results of his investigations. The measured, inductive tone of his memoirs might suggest that Knox was deliberately eschewing the type of speculation that Jameson indulged in in his lectures and private notes as to the place of the platypus in the natural order. Known for his flamboyant lecturing style and radical views, it could plausibly be suggested that in his published works at this time Knox wished to present himself as a hard-headed inductivist, scorning speculative opinions.

In this paper I have presented a case study showing how one particularly rare and interesting natural history specimen passed from a colonial governor in Australia, to the keeper of Edinburgh University's Museum, then to a renowned anatomist for dissection, and finally back to the keeper of the Museum to be added to its collection. In this case the creature concerned was also at the centre of a lively debate among the leading European comparative anatomists of the day. The question of how, if at all, the platypus could be fitted into existing taxonomic systems raised profound questions about the natural order. Such examples have much to teach about the political economy of natural history in the early nineteenth century, based on the circulation and exchange of both specimens and knowledge about the natural world. They also provide a window onto the way the possession and study of such specimens allowed existing models of the natural order to be confirmed or contested.

